# Surgical Quality, Antihypertensive Therapy, and Electrolyte Balance: A Novel Trifecta to Assess Long-Term Outcomes of Adrenal Surgery for Unilateral Primary Aldosteronism

**DOI:** 10.3390/jcm11030794

**Published:** 2022-02-01

**Authors:** Umberto Anceschi, Marilda Mormando, Cristian Fiori, Orazio Zappalà, Bernardino De Concilio, Aldo Brassetti, Alessandro Carrara, Maria Consiglia Ferriero, Gabriele Tuderti, Leonardo Misuraca, Alfredo Maria Bove, Riccardo Mastroianni, Alfonsina Chiefari, Marialuisa Appetecchia, Giuseppe Tirone, Francesco Porpiglia, Antonio Celia, Michele Gallucci, Giuseppe Simone

**Affiliations:** 1 Department of Urology, IRCCS “Regina Elena” National Cancer Institute, Via Elio Chianesi 53, 00144 Rome, Italy; aldo.brassetti@gmail.com (A.B.); marilia.ferriero@gmail.com (M.C.F.); gabriele.tuderti@gmail.com (G.T.); leonardo.misuraca@gmail.com (L.M.); alfredo.bove@ifo.gov.it (A.M.B.); riccardomastroianniroma@gmail.com (R.M.); michele.gallucci50@gmail.com (M.G.); puldet@gmail.com (G.S.); 2 Oncologic Endocrinology Unit, IRCCS “Regina Elena” National Cancer Institute, Via Elio Chianesi 53, 00144 Rome, Italy; marilda.mormando@ifo.gov.it (M.M.); alfonsina.chiefari@ifo.gov.it (A.C.); marialuisa.appetecchia@ifo.gov.it (M.A.); 3 Department of Urology, AOU San Luigi Gonzaga, Regione Gonzole, 10, 10043 Orbassano, Italy; cristian.fiori@unito.it (C.F.); francesco.porpiglia@unito.it (F.P.); 4 Department of General Surgery, Santa Chiara Regional Hospital, APSS, Largo Medaglie d’Oro 9, 38122 Trento, Italy; orazio.zappala@apss.tn.it (O.Z.); giuseppe.tirone@apss.tn.it (G.T.); 5 Department of Urology, San Bassiano Hospital, ULSS 7 Pedemontana, Via dei Lotti, 40, 36061 Bassano del Grappa, Italy; bernardinodeconcilio@hotmail.com (B.D.C.); antonio.celia@aulss7.veneto.it (A.C.); 6 Department of General Surgery, Santa Maria del Carmine Hospital, APSS, Corso Verona 4, 38068 Rovereto, Italy; alessandro.carrara@apss.tn.it

**Keywords:** trifecta, primary aldosteronism, adrenalectomy, PASO, Conn’s syndrome

## Abstract

Background: To propose a trifecta that summarizes endpoints and predicts their maintenance after adrenalectomy (*n* = 90) for unilateral primary aldosteronism (UPA). Methods: Trifecta was defined as coexistence of: ≥50% antihypertensive therapeutic intensity score reduction (∆TIS), no hypokalemia at 3 months, and no Clavien grade 2–5. Logistic regression was used to identify predictors of trifecta. Probability of clinical, biochemical, and simultaneous success according to trifecta were assessed by Kaplan–Meier. Cox regression was used to identify predictors of long-term clinical, biochemical, and simultaneous success. For all analyses, a two-sided *p* < 0.05 was considered significant. Results: Simultaneous success rate was 50%. On multivariable analysis, TIS was an independent predictor of trifecta achievement (HR 3.28; 95% CI 1.07–10.9; *p* = 0.03). At Kaplan–Meier, trifecta predicted higher success for all endpoints (each *p* < 0.03). On multivariable Cox analysis, adenoma size (AS) ≥6 cm and trifecta were independent predictors of biochemical (AS: HR 2.87; 95% CI 1.53–5.36; trifecta: HR 2.1; 95% CI 1.13–3.90; each *p* < 0.02) and simultaneous success (AS: HR 3.81; 95% CI 1.68–8.65; trifecta: HR 4.29; 95% CI 2.08–8.86; each *p* < 0.01), while trifecta was an independent predictor of complete clinical success (HR 2.84; 95% CI 1.45–5.58; *p* < 0.01). Conclusions: Trifecta and AS are independent predictors of either long-term complete clinical, biochemical, or combined success after adrenalectomy for UPA.

## 1. Introduction

Although recent surveys showed a higher trend of performance toward general surgeons, minimally-invasive adrenalectomy (MIA) remains a procedure of interest to urologists [1,2,3,4]. Pathologic evaluations from large adrenalectomy series describe unilateral primary aldosteronism (UPA) as the most common benign adrenal disease with an estimated incidence ranging between 5–13% in the general population [5,6,7].

The recent introduction of PASO criteria for describing clinical results of adrenalectomy for UPA did not obviate the historical variability in outcomes reporting with a significant proportion of patients (35–66%) experiencing either persistent hypertension (pHTN) or biochemical failure after surgery [8,9]. Although several predictive scores for the normalization of blood pressure after UPA treatment have been already conceived, a clinical tool for evaluating surgical quality and predicting long-term major endpoints of adrenalectomy for UPA has not yet been determined. [10,11,12]. In analogy to other major urological procedures, the concept of “trifecta” for standardizing adrenalectomy results in a single scoring system would represent a convenient tool to describe and predict main outcomes as to increase reproducibility between series [13,14,15]. 

In this scenario, we sought to develop a novel scoring system for standardizing UPA surgical management and to assess its ability to predict clinical, biochemical, and combined success at an extended follow-up on a multi-institutional, minimally-invasive, adrenalectomy dataset.

## 2. Material and Methods

Between March 2011 and March 2021, a collaborative, board-approved, multicentric dataset was queried for “unilateral primary aldosteronism (UPA)” and “partial” and “total adrenalectomy.” Exclusion criteria were considered as following: any patient with bilateral adrenal disease or any malignant adrenal disease (primitive or metastatic) with missing perioperative data and/or follow-up <18 months. In all patients, UPA was associated to a single, unilateral adrenal mass diagnosed on computed tomography (CT) or magnetic resonance imaging (MRI) or adrenal venous sampling (AVS), according to each center’s diagnostic work-up and availability. Indications for MIPA were limited to adrenal masses <3 cm. 

Clinical diagnosis of UPA was confirmed by oral sodium loading test in 17 patients (18.8%), saline infusion test in 60 patients (66.7%), fludrocortisone suppression test in 8 (8.9%), and captopril challenge test in 5 (5.6%), respectively. UPA was confirmed by AVS in 38 patients (42.2%). According to Endocrine Society guidelines, for patients <35 years old with spontaneous hypokalemia and unilateral adrenal lesions with radiological features consistent with a cortical adenoma on adrenal CT/MRI scan, confirmatory AVS was not deemed necessary in 4.4% of cases. [16,17]. Furthermore, other causes of adrenal-related endocrine HTN (eHTN), such as Cushing’ syndrome or pheochromocytoma, were excluded in all patients. More in detail, hypersecretion of catecholamines was investigated through 24-h urine collection for fractionated metanephrines as ACTH-independent cortisol excess by 1 mg dexamethasone suppression test [18,19].

A total of 90 eligible patients were identified. Follow-up schedule was similar among centers, including an endocrinologic evaluation at 3, 6, and 12 months after surgery, while a cardiologist was scheduled in case of persistent hypertension. Demographic, preoperative and perioperative data, pathological outcomes, and follow-up data were retrospectively reviewed. 

Information on antihypertensive therapy (number of drugs, class, and daily dose) were retrieved from clinical charts for each patient and computed by TIS metric. It represents a proportional measure of prescribed to maximum U.S. Food and Drug Administration (FDA) recommended dosage that was calculated for each antihypertensive medication [20,21]. The list of drugs used in the cohort for each TIS score assignment is reported in Table 1.

Individual TIS scores of each antihypertensive medication were calculated for each patient and incorporated into a single, cumulative TIS score preoperatively and at 3 months follow-up in order to estimate the ΔTIS = [(Preoperative TIS score—TIS score at 3 months)/preoperative TIS score] × 100.

The following preoperative data were analyzed: age, gender, American Society of Anesthesiology (ASA) score, preoperative hemoglobin (Hb), TIS score, adenoma size (AS) and side, and serum potassium level. Intraoperative variables included mean operative time (MOT), surgical approach (partial/total), % perioperative complications, and % perioperative transfusions. Postoperative parameters of interest were length of hospital stay (LOS), postoperative Hb, and median perioperative Hb drop (ΔHb). Complications within 30 days after surgery were recorded and standardized according to the Clavien–Dindo system. [22]. Long-term functional results were retrieved and stratified according to Primary Aldosteronism Surgical Outcomes (PASO) criteria. [8] More in detail, a complete clinical success was defined as normal blood pressure without the aid of antihypertensive medication, while a partial clinical success was defined as the same blood pressure as before surgery with less antihypertensive medication or a reduction in blood pressure with either the same amount or less antihypertensive medication. Absent clinical success was defined as unchanged or increased blood pressure with either the same amount or an increase in antihypertensive medication. Complete biochemical success was defined as correction of hypokalemia (if present prior to surgery), while persistent hypokalemia after adrenalectomy was considered as an absent biochemical success. The rate of cortisol replacement for each group was also reported.

Data were used to outline two binary variables for the achievement of trifecta (defined as the contemporary absence of significant perioperative complications (any Clavien-Dindo Grade 2–5, ΔTIS reduction ≥ 50%, and no hypokalemia at 3 months after surgery). Primary endpoints of the study were to determine the trifecta rates between groups and to identify predictors of its achievement in the overall cohort. Differences between continuous variables were assessed with Wilcoxon rank-sum test, while Pearson’s χ^2^ test was used for categorical data. Probability of complete clinical, biochemical, and simultaneous success according to standardized Primary Aldosteronism Surgical Outcomes criteria (PASO) were assessed by Kaplan–Meier method and compared with the log-rank test. Univariable and multivariable logistic regression analysis were used to identify predictors of trifecta achievement. 

The secondary endpoint was to determine the ability of trifecta in predicting long-term, complete clinical, biochemical, and combined success. Univariable and multivariable Cox regression analyses were used to identify predictors of long-term functional outcomes. For all analyses, a two-sided *p* < 0.05 was considered significant. Statistical analysis was carried out using Statistical Package for Social Sciences (SPSS) software v.26.0 (IBM Corp, Armonk, NY, USA).

## 3. Results

A total of 90 consecutive patients with a confirmed diagnosis of UPA were identified among centers. Baseline characteristics of the cohort according to surgical indication are shown in Table 2.

No differences was reported between groups in terms of any parameter considered (each *p* > 0.2) except for median tumor size, which was significantly lower in MIPA cohort. (*p* = 0.001). Preoperative hypertension rate was similar among series (86.8% vs. 93.2%; *p* = 0.456) as the median cumulative TIS score (range 0.25–1; *p* = 0.98), with up to one-third of the overall cohort requiring a combined class of medications (*p* = 0.676). Preoperative hypokalemia rates were negligible between series (*p* = 0.184). 

Perioperative and pathologic outcomes are summarized in Table 3.

Although no difference was found between groups in terms of ΔHb (*p* = 0.337), median LOS was slightly increased in the MITA series (4 d vs. 3 d; *p* = 0.038). The overall complications rate was 11.1% with the following distribution: grade I–II complications, 10%; grade III–V complications, 1.1%. Perioperative transfusions rate was comparable between groups (3.2% vs. 3.4%; *p* = 0.967). Final pathology revealed a capsulated adenoma in 77.8% of cases, while 22.2% of patients showed diffuse adrenal hyperplasia (*p* = 0.209). 

Early (3 months) and long-term functional outcomes are reported in Table 4 and Table 5.

The overall trifecta rate was 21.1%. In the MITA group, the trifecta was 16.3% (*n* = 10) with 13 (21.3%) patients showing ΔTIS ≥ 50%, 53 (86.8%) reporting no major complications, and 51 patients (83.6%) with no electrolyte imbalance respectively, while in the MIPA cohort, the trifecta rate was 31% with 12 patients (41.3%) achieving a ΔTIS ≥ 50%, 27 patients (93.1%) reporting no major complications, and 25 patients (86.2%) with absence of hypokalemia. At a median follow-up of 42 months, complete, partial, and absent clinical success rates were achieved in 54 (60%), 16 (17.7%), and 20 (22.3%) patients, respectively, while a complete, partial, and absent biochemical success was obtained in 75 (83.3%), 11 (12.3%), and 4 (4.4%) patients, respectively. Overall, hypokalemia was observed in 13.3% of patients.

On multivariable logistic regression analysis, preoperative TIS ≥ 0.5 score (OR 3.28; 95% CI 1.07–10.9; *p* = 0.03) was the only independent predictor of trifecta achievement (Table 6). 

**Figure 1:**
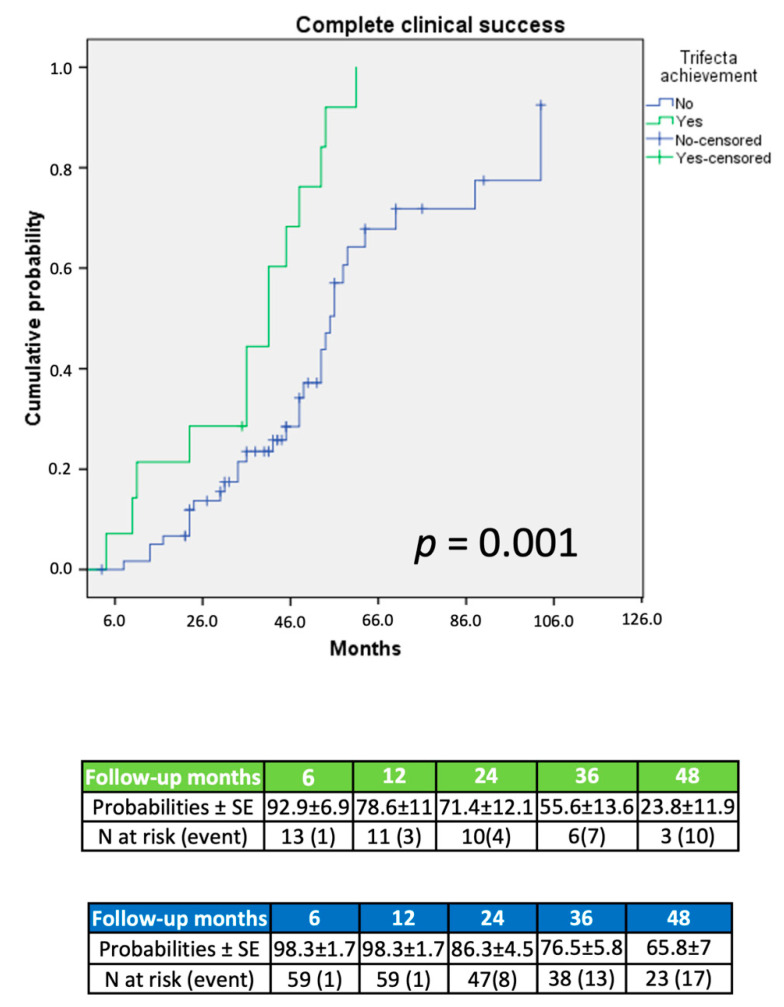
Probability of complete clinical success according to PASO criteria.

**Figure 2:**
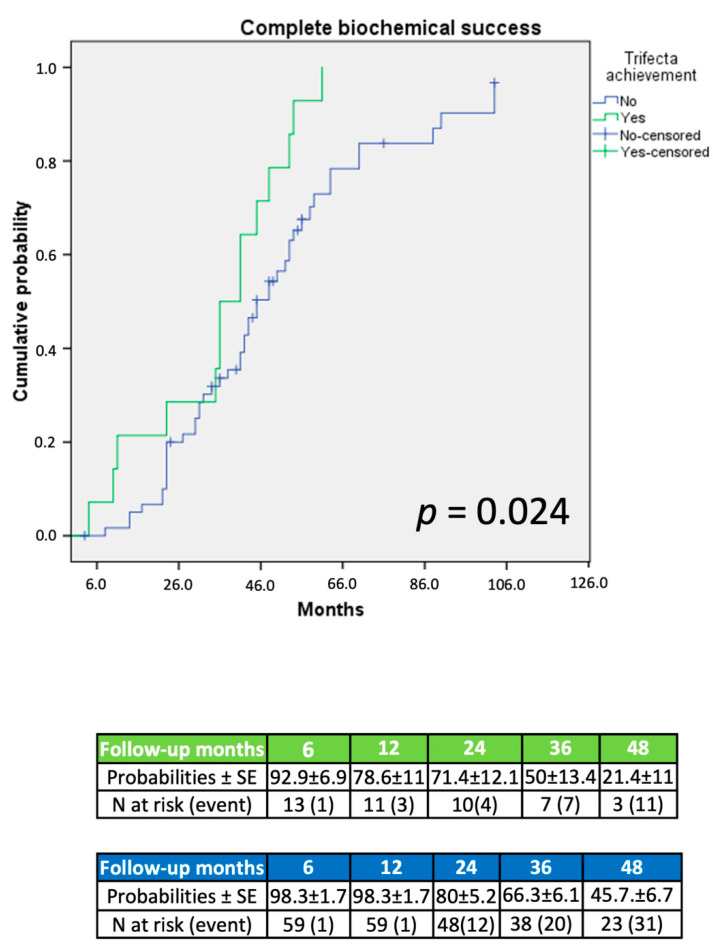
Probability of complete biochemical success according to PASO criteria.

**Figure 3:**
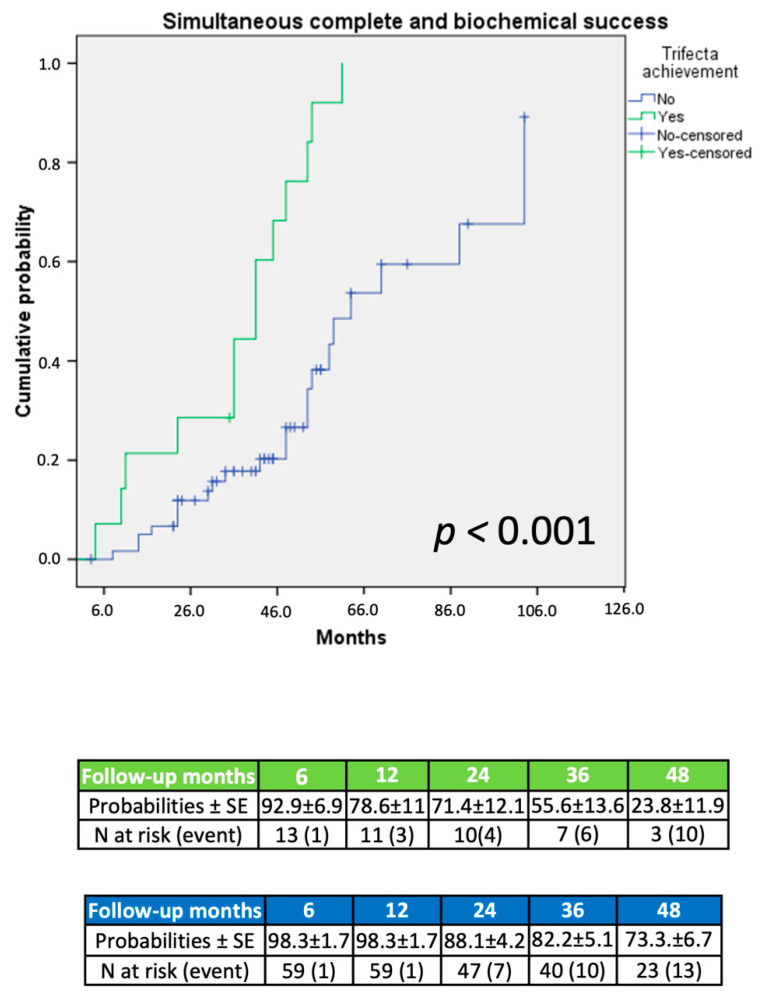
Probability of simultaneous complete clinical and biochemical success according to PASO criteria.

At Kaplan–Meier analysis, patients achieving trifecta displayed significantly higher complete clinical (*p* = 0.001; Figure 1), biochemical (*p* = 0.02; Figure 2), and simultaneous success (*p* < 0.001; Figure 3) rates at an extended follow-up, respectively. 

On multivariable Cox regression analysis, trifecta achievement (HR 2.84; 95% CI 1.45–5.58; *p* = 0.002) was an independent predictor of complete clinical success, while AS ≥ 6 cm (HR 2.87; 95% CI 1.53–5.36; *p* = 0.001) and trifecta (HR 2.10; 95% CI 1.13–3.90; *p* = 0.018) were independent predictors of complete biochemical success. AS ≥ 6 cm (HR 3.81; 95% CI 1.68–8.65; *p* < 0.001) and trifecta achievement (HR 4.29; 95% CI 2.08–8.06; *p* < 0.001) were also both predictors of synchronous complete and biochemical success in the long-run (Table 7, Table 8 and Table 9).

## 4. Discussion

According to PASO criteria, the standardized composite outcome of adrenalectomy for UPA is represented by the concurrent attainment of normalization of blood pressure without the aid of antihypertensive medication and the absence of hypokalemia [8]. Even though the synchronous achievement of complete clinical and biochemical success remains a goal restricted to a minority of patients affected by UPA, the stiff thresholds considered as the intrinsic dichotomy of PASO criteria did not improve the historical heterogeneity in adrenalectomy outcomes reporting [5,9]. Since the incidence of electrolyte imbalance after adrenal gland removal remains negligible in the UPA setting, the decrease of blood pressure and/or the number of antihypertensive drugs remain the primary endpoint in most of contemporary adrenalectomy series [23,24]. Consequently, the rate of combined clinical and biochemical endpoints in the single patient are not supported by current literature [25,26]. Furthermore, whether the early achievement of PASO criteria after surgery may avoid a later onset of pHTN or hypokalemia remains unclear. [5] Recently, Vorselaars et al. introduced the concept of “clinical cure,” defining as clear improvement the normalization of blood pressure receiving a lower or equal number of antihypertensive medications [25,26,27]. The application of this system, however, could generate further issues since it provides neither clear information on antihypertensive dosage nor the evaluation of an eventual biochemical failure. 

To overcome these limitations, herein, we proposed a familiar method of outcomes analysis to urologists, called the “trifecta,” which incorporates the following three main objectives of adrenalectomy for UPA resolution: the absence of perioperative complications, the blood pressure control (defined as a total or critical decrease of dose and number of antihypertensive medications), and the preservation of electrolyte balance. In this scenario, we customized a novel score to provide a widely reproducible tool based on standardized parameters that comprehensively summarizes early adrenalectomy outcomes regardless of surgical approach considered. Moreover, we tested its implications in the long term.

The ΔTIS ≥ 50% was used as proxy for blood pressure control (BPC) [20,21]. This algorithm provides a quick and detailed antihypertensive dosing intensity metric compared to the raw number of prescribed drugs or daily doses usually reported in major adrenalectomy series [23,24]. This surrogate parameter cannot replace entirely blood pressure normalization; however, obtaining a significant decrease of use of antihypertensive medications represents an obvious goal of adrenalectomy for UPA regardless of its quantifiable effect. In our series, ΔTIS ≥50% was observed in 25 patients (27.8%). Comparable clinical results in terms of decrease of use of antihypertensive medications were reported by other authors in major series [26,28].

Although associated with low mortality rates in high-volume centers, the description of perioperative adrenalectomy outcomes remains limited to population-based series [29]. Complications rates after adrenalectomy for UPA may be as high as 5%, and most of them are related to major comorbidities [30]. Therefore, in contrast with other urological procedures, herein, we adopted a stricter definition for trifecta achievement, excluding all patients who experienced a either medical or surgical significant complication (CD ≥ 2). In the present series, these occurred in 3.4% of the cases. Our rate was in line with previously published series on adrenalectomy [26,27]. 

Since the incidence of biochemical failure remains negligible if compared to the rate of partial or absent clinical success (16.7%), we endorsed the absence of electrolyte imbalance with the goal of defining the optimal adrenal function preservation, as persistent hypokalemia may represent an indicator of underestimated bilateral UPA, a side-effect of multidrug antihypertensive therapy, or a consequence of long-standing untreated UPA [31,32]. In our cohort, early hypokalemia occurred in 14 patients (15.6%). These findings are similar to those reported from multi-institutional international cohorts [8,26,27]. 

According to our highly restrictive definition, in the present series, only 19 patients achieved trifecta (21.1%). Undoubtedly, no comparison can be done with previous metrics since the clinical cure, as forementioned, does not consider the biochemical outcomes, while PASO criteria discriminate between three different subgroups but for dichotomized endpoints. Nonetheless, our trifecta rates seem comparable to clinical success rates reported in major series [11,12,27,28]. 

After adjusting our model for age, gender, adenoma size, surgical approach, and preoperative TIS score, on logistic univariable regression analysis, age (*p* = 0.04), AS (*p* = 0.04) and TIS ≥ score (*p* = 0.03) were significantly associated with trifecta achievement, while on multivariable analysis, TIS score ≥ 0.5 was the only independent predictor of trifecta achievement. As prior studies have described an established association between the number of preoperative drugs and normalization of blood pressure after adrenalectomy, it is reasonable to assume that higher preoperative TIS scores seem to be representative of patients affected by uncontrolled hypertension and, indirectly, with higher probability of early functional recovery after surgery [5,28]. Moreover, the relationship between age, adenoma size, and trifecta achievement on univariable analysis may remain clinically insignificant in the absence of major comorbidities, or it may represent a consequence of a regression model overfitting in our series (Table 4). 

On Kaplan–Meier analysis, trifecta achievement was associated to a significantly increased probability of clinical, biochemical, and simultaneous complete success (each *p* < 0.03; Figure 1, Figure 2 and Figure 3) at an extended follow-up (range: 27–54 months). These findings were confirmed at Cox regression analysis. Notably, after adjusting the regression model for age, gender, AS, surgical approach, and trifecta achievement, on multivariable analysis, trifecta and AS were the only independent predictors of any endpoint considered. More in detail, trifecta achievement and AS ≥ 6 cm were significant predictors of either complete biochemical or combined success (each *p* < 0.02), while only trifecta was predictor of complete clinical success (*p* = 0.01). In our cohort, larger AS seemed to be characterized by a more pronounced aldosterone secretion and higher probability of a full recovery after treatment; however, we cannot entirely rule out potential residual confounding from biochemical and clinical variables during follow-up not included in our dataset.

We acknowledge limitations in our study. Firstly, given the retrospective and multicentric nature of this dataset, our data are likely to have been subjected to selection, indication, and performance bias. Additionally, since BPC and electrolyte impairment could significantly be affected by major comorbidities, type of measurement, and duration of hypertension, we were unable to retrieve this information from our series. Furthermore, the lack of inclusion and assessment of target-organ damage related to long-standing UPA in the trifecta score may undoubtedly decrease its long-term predictive accuracy [33,34]. 

We are aware that an ideal score for UPA should discriminate between either partial or absent clinical and biochemical outcomes in order to identify which patients may potentially expect a prolonged improvement of BPC or avoid a later onset of hypokalemia. Additionally, the lack of external validation of this scoring system enhances the risk of an inconsistent pattern. Nonetheless, according to our results, trifecta represent an easy clinical triad through which physicians may standardize outcomes reporting and predict long-term endpoints of adrenalectomy for UPA at once.

## 5. Conclusions

Trifecta achievement and AS ≥ 6 cm represent significant prognostic factors by which physicians may counsel patients before adrenalectomy on the probability of a long-term simultaneous and/or biochemical cure. We proposed the introduction of a new algorithm aimed at standardizing early adrenalectomy outcomes for UPA in order to provide maintenance of blood pressure control, electrolyte balance, and surgical quality over time.

## Figures and Tables

**Table 1:** List of preoperative drugs used in the cohort for the TIS score assessment.

Type of Drug	Minimum Dosage (Hypertension)	Maximum Dosage (Hypertension)
Zofenopril	7.5	60
Nebivolol	5	10
Ramipril	2.5	10
Nifedipine	30	60
Enalapril	5	40
Manidipine	10	20
Doxazosine	2	16
Furosemide	25	1000
Indapamide	1	5
Delapril	15	60
Irbesartan	150	300
Losartan	12.5	150
Hydrochlorothiazide	12.5	200
Clonidine	75	900
Carvedilol	6.25	50
Atenolol	25	100
Amlodipine	5	10
Valsartan	40	320
Olmesartan	5	40
Lercanidipine	10	30
Aldactone	25	400
Canrenone	50	800
Barnidipine	5	20
Pentoxyfilline	400	1200
Bisoprolol	2.5	10

**Table 2:** Baseline and preoperative data.

Variable	Overall Cohort	Total Adrenalectomy	Partial Adrenalectomy	*p*
Age at surgery (median, IQR)	54 (44–65)	54 (44.5–63)	57 (43.5–67.5)	0.408
Follow-up (months, median, range)	42 (27–54)	41 (24–50)	46 (32.7–57.5)	0.223
Gender (*n*, %)				
Male	36 (40%)	23 (37.7%)	13 (44.8%)	
Female	54 (60%)	38 (62.3%)	16 (55.2%)	0.519
ASA score (*n*, %)				
1–2	73 (81.1%)	50 (82%)	23 (79.3%)	
3–4	17 (18.9%)	11 (18%)	6 (20.7%)	0.763
Adrenal mass size (cm, *n*, IQR)	3 (2–5)	4.2 (2.35–6)	2.7 (1.8–2.85)	0.001
Side (*n*, %)				
Left	45 (50%)	23 (37.7%)	22 (75.9%)	
Rigth	45 (50%)	38 (62.3%)	7 (24.1%)	0.001
Preoperative Hypertension (*n*, %)				
Yes	80 (88.8%)	53 (86.8%)	27 (93.1%)	
No	10 (11.2%)	8 (13.2%)	2 (6.9%)	0.456
Preoperative Hypokalemia (*n*,%)				
Yes	27 (30%)	21 (65.6%)	6 (20.7%)	
No	63 (70%)	40 (34.4%)	23 (79.3%)	0.184
Number of drugs (*n*,%)				
No drugs	9 (10%)	7 (11.4%)	2 (6.8%)	
One class medication	50 (55.5%)	32 (52.4%)	18 (62%)	
Combined class medication (≥2)	31 (34.5%)	22 (36%)	9 (31.2%)	0.676
Preoperative TIS score (median, IQR)	0.5 (0.25–1)	0.5 (0.25–1.09)	0.5 (0.25–1)	0.989

**Table 3:** Perioperative and pathologic outcomes.

Variable	Overall Cohort	Total Adrenalectomy	Partial Adrenalectomy	*p*
Preoperative Hb (g/dL, median, IQR)	13.8 (12.8–14.6)	13.4 (12.5–14.3)	14.3 (13.4–14.9)	0.058
Postoperative Hb (g/dL, median, IQR)	12.6 (11.7–13.5)	12.3 (11.6–13.4)	13.3 (11.7–13.5)	0.271
ΔHb (g/dL, median, IQR)	1.1 (0.3–2.1)	1.1 (0.1–1.8)	1.1 (0.4–2.35)	0.337
LOS (days, median, IQR)	4 (3–5)	4 (3–5)	3 (2.5–4)	0.038
Overall complications (*n*, %)	10 (11.1%)	7 (11.5%)	3 (10.3%)	0.873
Perioperative transfusions rate (*n*, %)	3 (3.4%)	2 (3.2%)	1 (3.4%)	0.967
Clavien Grade (*n*, %)				
I	6	6	2	0.940
II	3	2	1	
III	-	-	-	
IV	1	1	-	
V	-	-	-	0.488
Follow-up (months, median range)	42 (27–54)	41 (24–50)	46 (32.7–57.5)	0.223
Histology (*n*, %)				
Adenoma	70 (77.8%)	48 (78.7%)	22 (75.8%)	
Hyperplasia	20 (22.2%)	13 (21.3%)	7 (24.1%)	0.209

**Table 4:** Early and long-term functional outcomes after MIPA or MITA.

Variable	Overall Cohort	Total Adrenalectomy	Partial Adrenalectomy	*p*
Trifecta	19 (21.1%)	10 (16.3%)	9 (31%)	0.312
- 3-month ∆TIS ≥ 50%	25 (27.8%)	13 (21.3%)	12 (41.3%)	0.067
- No CKD ≥ 2	80 (88.9%)	53 (86.8%)	27 (93.1%)	0.813
- No hypokalemia (3 months)	76 (84.4%)	51 (83.6%)	25 (86.2%)	0.837
Follow-up (months, median, IQR)	42 (27–54)	41 (24–50)	46 (32.7–57.5)	0.223
Hypokalemia at last follow-up (*n*, %)	12 (13.3%)	9 (14.8%)	3 (10.3%)	0.565
Cortisol replacement at last follow-up (*n*, %)	4 (6.5%)	4 (6.5%)	-	-
Complete clinical success (*n*, %)	54 (60%)	33 (54%)	21 (72.4%)	0.097
Partial clinical success (*n*, %)	16 (17.7%)	14 (23%)	2 (6.8%)	0.136
Absent clinical success (*n*, %)	20 (22.3%)	14 (23%)	6 (20.7%)	0.136
Complete biochemical success (*n*, %)	75 (83.3%)	50 (81.9%)	25 (86.2%)	0.918
Partial biochemical success (*n*, %)	11 (12.3%)	7 (11.4%)	4 (13.7%)	0.918
Absent biochemical success (*n*, %)	4 (4.4%)	3 (4.91%)	1 (3.4%)	0.918

**Table 5:** Early and long-term functional outcomes according to PASO criteria.

Variable	Overall Cohort	Total Adrenalectomy	Partial Adrenalectomy	*p*
Complete clinical success				
- No medication/Controlled BP	54 (60%)	33 (54%)	21 (72.4%)	0.097
Partial clinical success	16 (17.7%)	14 (23%)	2 (6.8%)	
- Drug escalation (Controlled BP)	8 (8.9%)	7 (11.5%)	1 (3.4%)	
- Switch to a lower class of medication (Controlled BP)	2 (2.2%)	2 (3.3%)	-	
- No drugs (Moderate BP reduction)	4 (4.4%)	4 (6.6%)	-	0.136
- Switch to comparable medication (Moderate BP reduction)	2 (2.2%)	1 (1.6%)	1 (3.4%)	
Absent clinical success	20 (22.3%)	14 (23%)	6 (20.7%)	
- Unchanged dosage medication	14 (15.6%)	9 (14.8%)	5 (17.2%)	
- Increased dosage	3 (3.3%)	3 (4.9%)	-	0.136
- Switch to a stronger class of medication	3 (3.3%)	2 (3.3%)	1 (3.4%)	

**Table 6:** Univariable and multivariable logistic regression analysis to identify predictors of trifecta achievement.

Variable	Univariable Analysis				Multivariable Analysis			
	OR	95.0% CI			OR	95.0% CI		
		Lower	Higher	*p*-Value		Lower	Higher	*p*-Value
Age	3.44	1.04	11.4	0.04	3.07	0.88	10.6	0.07
Gender	1.32	0.46	3.77	0.594	-	-	-	-
ASA score								
1–2	0.76	0.19	2.98	0.698	-	-	-	-
3–4								
Adenoma size (cm)	1.18	1.02	1.39	0.04	1.14	0.95	1.36	0.152
Surgical approach	1.31	0.37	4.64	0.675	-	-	-	-
TIS score (0.5≤ vs. ≥0.5)	3.53	1.21	10.3	0.02	3.28	1.07	10.9	0.03

**Table 7:** Univariable and multivariable logistic regression analysis to identify predictors of complete clinical success according to PASO criteria.

Variable	Univariable Analysis				Multivariable Analysis			
	HR	95.0% CI			HR	95.0% CI		
		Lower	Higher	*p*-Value		Lower	Higher	*p*-Value
Age (≤50 year vs. ≥50 year)	0.81	0.44	1.47	0.501	-	-	-	-
Gender	1.18	0.62	2.26	0.596	-	-	-	-
ASA score								
1–2	0.65	0.30	1.38	0.262	-	-	-	-
3–4								
Adenoma size (≤6 cm vs. ≥6 cm)	1.12	1.01	1.24	0.03	1.16	0.99	1.24	0.05
MIPA vs. MITA	1.63	0.67	3.95	0.276	-	-	-	-
Perioperative complications (CD II–V)	0.18	0.02	1.34	0.09				
ΔTIS reduction ≥ 50	2.47	1.3	4.69	0.006				
No Hypokalemia (3 months)	0.70	0.33	1.47	0.350				
Trifecta	2.96	1.56	5.82	0.002	2.84	1.45	5.58	0.002

**Table 8:** Univariable and multivariable logistic regression analysis to identify predictors of complete biochemical success according to PASO criteria.

Variable	Univariable Analysis				Multivariable Analysis			
	HR	95.0% CI			HR	95.0% CI		
		Lower	Higher	*p*-Value		Lower	Higher	*p*-Value
Age (≤50 y vs. ≥50 y)	1	0.98	1.02	0.738	-	-	-	-
Gender	1.30	0.75	2.26	0.336	-	-	-	-
ASA score								
1–2	0.68	0.36	1.28	0.237	-	-	-	-
3–4								
Adenoma size (≤6 cm vs. ≥ 6 cm)	2.72	1.46	5.06	0.001	2.87	1.53	5.36	0.001
MIPA vs. MITA	1.27	0.74	2.18	0.370	-	-	-	-
Perioperative complications (CD II–V)	1	0.45	2.23	0.984				
ΔTIS reduction ≥ 50%	1.20	0.66	2.19	0.546				
No Hypokalemia (3 months)	0.25	0.10	0.64	0.004				
Trifecta	1.97	1.07	3.65	0.03	2.10	1.13	3.90	0.018

**Table 9:** Univariable and multivariable logistic regression analysis to identify predictors of simultaneous complete clinical and biochemical success according to PASO criteria.

Variable	Univariable Analysis				Multivariable Analysis			
	HR	95.0% CI			HR	95.0% CI		
		Lower	Higher	*p*-Value		Lower	Higher	*p*-Value
Age (≤50 y vs. ≥50 y)	0.95	0.49	1.85	0.890	-	-	-	-
Gender	1.44	0.69	3.01	0.326	-	-	-	-
ASA score								
1–2	0.48	0.19	1.19	0.114	-	-	-	-
3–4								
Adenoma size (≤6 cm vs. ≥ 6 cm)	2.50	1.08	5.79	0.032	3.81	1.68	8.65	0.001
MIPA vs. MITA	1.97	0.79	4.86	0.141	-	-	-	-
Perioperative complications (CD II–V)	0.22	0.03	1.62	0.137				
ΔTIS reduction ≥ 50%	2.2	1.07	4.49	0.031				
No Hypokalemia (3 months)	0.34	0.12	0.97	0.045				
Trifecta	4.10	2.01	8.43	<0.001	4.29	2.08	8.86	<0.001

## Data Availability

The data presented in this study are available on request from the corresponding author. The data are not publicly available due to privacy issue.
